# Damaging variants in *FOXI3* cause microtia and craniofacial microsomia

**DOI:** 10.1016/j.gim.2022.09.005

**Published:** 2022-10-19

**Authors:** Daniel Quiat, Andrew T. Timberlake, Justin J. Curran, Michael L. Cunningham, Barbara McDonough, Maria A. Artunduaga, Steven R. DePalma, Milagros M. Duenas-Roque, Joshua M. Gorham, Jonas A. Gustafson, Usama Hamdan, Anne V. Hing, Paula Hurtado-Villa, Yamileth Nicolau, Gabriel Osorno, Harry Pachajoa, Gloria L. Porras-Hurtado, Lourdes Quintanilla-Dieck, Luis Serrano, Melissa Tumblin, Ignacio Zarante, Daniela V. Luquetti, Roland D. Eavey, Carrie L. Heike, Jonathan G. Seidman, Christine E. Seidman

**Affiliations:** 1 Department of Cardiology, Boston Children’s Hospital, Boston, MA; 2 Department of Pediatrics, Harvard Medical School, Boston, MA; 3 Department of Genetics, Harvard Medical School, Boston, MA; 4 Hansjörg Wyss Department of Plastic and Reconstructive Surgery, NYU Langone Medical Center, New York, NY; 5 Division of Craniofacial Medicine, Department of Pediatrics, University of Washington, Seattle, WA; 6 Center for Developmental Biology and Regenerative Medicine, Seattle Children’s Research Institute, Seattle, WA; 7 Respira Labs, Inc, Mountain View, CA; 8 Hospital Edgardo Rebagliati Martins, EsSalud, Lima, Peru; 9 Global Smile Foundation, Norwood, MA; 10 Pontificia Universidad Javeriana and Centro Médico Imbanaco, Cali, Colombia; 11 Texas ENT Specialists, Houston, TX; 12 Facultad de Medicina, Universidad Nacional de Colombia, Bogotá, Colombia; 13 Servicio de Genética Médica, Fundación Valle del Lili, Cali, Colombia; 14 Centro de Investigación en Anomalías Congénitas y Enfermedades Raras (CIACER), Universidad Icesi, Cali, Colombia; 15 Clinica Comfamiliar Risaralda, Pereira, Colombia; 16 Department of Otolaryngology Head and Neck Surgery, Oregon Health & Science University, Portland, OR; 17 Audiocentro, Cuenca, Ecuador; 18 Ear Community, Inc, Broomfield, CO; 19 Human Genomics Institute, Pontificia Universidad Javeriana, Bogotá, Colombia; 20 Hospital Universitario San Ignacio, Bogotá, Colombia; 21 Department of Otolaryngology–Head & Neck Surgery, Vanderbilt University Medical Center, Nashville, TN; 22 Cardiovascular Division, Brigham and Women’s Hospital, Boston, MA; 23 Howard Hughes Medical Institute, Chevy Chase, MD

**Keywords:** Craniofacial microsomia, Ectoderm, FOXI3, Microtia, Neural crest

## Abstract

**Purpose:**

Craniofacial microsomia (CFM) represents a spectrum of craniofacial malformations, ranging from isolated microtia with or without aural atresia to underdevelopment of the mandible, maxilla, orbit, facial soft tissue, and/or facial nerve. The genetic causes of CFM remain largely unknown.

**Methods:**

We performed genome sequencing and linkage analysis in patients and families with microtia and CFM of unknown genetic etiology. The functional consequences of damaging missense variants were evaluated through expression of wild-type and mutant proteins in vitro.

**Results:**

We studied a 5-generation kindred with microtia, identifying a missense variant in *FOXI3* (p.Arg236Trp) as the cause of disease (logarithm of the odds = 3.33). We subsequently identified 6 individuals from 3 additional kindreds with microtia-CFM spectrum phenotypes harboring damaging variants in *FOXI3*, a regulator of ectodermal and neural crest development. Missense variants in the nuclear localization sequence were identified in cases with isolated microtia with aural atresia and found to affect subcellular localization of FOXI3. Loss of function variants were found in patients with microtia and mandibular hypoplasia (CFM), suggesting dosage sensitivity of *FOXI3*.

**Conclusion:**

Damaging variants in *FOXI3* are the second most frequent genetic cause of CFM, causing 1% of all cases, including 13% of familial cases in our cohort.

## Introduction

The microtia-craniofacial microsomia (CFM) spectrum of disorders (OMIM 164210, 251800) is the second most common congenital facial anomaly, with estimations of prevalence ranging from 1 in every 3000 to 5000 live births.^1^ Although genes implicated in neural crest cell migration and patterning, chromatin modification, fibroblast growth factor receptor signaling, ribosome assembly, and the spliceosome have been implicated in monogenic syndromes that include malformations of the ears or mandible,^2^ most cases are sporadic and of unknown genetic etiology. Previous genetic studies employing exome and genome sequencing (GS) of CFM trios confirm substantial locus heterogeneity.^3^

## Materials and Methods

### Sample populations

Two independently ascertained cohorts were used for this study, which we denote Gabriella Miller Kids First (GMKF) CFM and GMKF-microtia. For the GMKF-CFM cohort, we enrolled families with CFM in our multinational research consortium from 2009 to 2020. Individuals aged between 0 and 18 years were eligible for the study if they met at least one of the following inclusion criteria: (1) microtia or anotia, (2) mandibular hypoplasia and preauricular tag, (3) mandibular hypoplasia and facial tag, (4) mandibular hypoplasia and epibulbar dermoid, (5) mandibular hypoplasia and lateral oral cleft (eg, macrostomia), (6) preauricular tag and epibulbar dermoid, (7) preauricular tag and lateral oral cleft, (8) facial tag and epibulbar dermoid, and (9) lateral oral cleft and epibulbar dermoid and consenting parent spoke a language in which they were eligible for consent at their enrolling site. Individuals were excluded if they had an abnormal karyotype or a syndromic diagnosis that involves microtia or underdevelopment of the jaw. DNA was extracted from blood and saliva using standard procedures (Qiagen and Oragene kits).

For GMKF-microtia cohort patients, unaffected relatives were identified at collaborating sites in South America and the United States as previously described.^4^ Subjects were selected for isolated microtia in the absence of syndromic features, and patients with syndromic forms of microtia were excluded from the study. Peripheral blood samples were collected for DNA extraction.

### Exome sequencing and GS

Exome sequencing was performed at either the Northwest Genomics Center (University of Washington) or GENEWIZ using the Nimblegen SeqCap Human Exome v2.0 or Agilent SureSelect Human All Exon capture platforms, respectively. GS was performed at the Broad Institute or the Northwest Genomics Center, with sequence data generated on the Illumina HiSeq X platform.

### Variant analyses

For GMKF-CFM variants were called using the Genome Analysis Toolkit pipeline (Poplin R, Ruano-Rubio V, DePristo MA, et al. Scaling accurate genetic variant discovery to tens of thousands of samples. 2017:201178. https://doi.org/10.1101/201178) and annotated using ANNOVAR, with allele frequencies derived from the Genome Aggregation Database (gnomAD).^5^ For GMKF-microtia subjects, variants were called as previously described.^4^ *FOXI3* variants were validated through bidirectional Sanger sequencing of the proband and parents.

### Identification of shared haplotypes in kindred 1

We filtered GS variant calls for biallelic single nucleotide variants (SNVs) with variant quality score recalibration log-odds Filter PASS (truth sensitivity 99.8%), depth of >10, genotype quality of >60, and gnomAD (v3) non-Finnish European allele frequency (AF) of <1e-05. Variants were further filtered to obtain only those SNVs in a heterozygous state in all affected individuals (BCFtools).

### Linkage analysis

We used Mega2 (v6.0.0) to format genotype and phenotype data for parametric linkage analysis in Simwalk2 (v2.91), accounting for the observed penetrance of 30% and an estimate population AF of 1e-05. Simwalk2 reports a location score that is directly comparable to multipoint logarithm of the odds (LOD) score and as such is reported as a LOD score in the text.

### Burden analysis

We compared the frequency of variants (loss of function [LOF] or missense in nuclear localization sequence [NLS]) identified in the microtia-CFM cohort with the number in gnomAD v3.1.2 using the median allele number across all *FOXI3* variant positions (151,991) using a Fisher exact test. Variants in low complexity regions and conservative missense variants (Arg to His) were excluded from the analysis.

### Generation of FOXI3 expression plasmids

A previously reported human FOXI3 expression plasmid containing an N-terminal FLAG-tag^6^ was purchased from Addgene (catalog number 153128). We performed site-directed mutagenesis using the Agilent Quikchange II XL kit (catalog number 200521) according to manufacturer specification to introduce missense variants. Wild-type and mutant construct sequences were confirmed through Sanger sequencing.

### Immunocytochemistry

HEK293 cells (92A001, CH3 Biosystems) cultured in Dulbecco’s Modified Eagle Medium (11995–073, Gibco) supplemented with 10% fetal bovine serum (A3840202, Gibco) were used for transfection. Cells were grown to approximately 20% confluency, and then, wild-type and mutant FLAG-FOXI3 expression plasmids (2 ug each) were transfected using Lipofectamine 3000 (L3000001, ThermoFisher Scientific). Immunocytochemistry was performed using a monoclonal mouse anti-FLAG antibody (F1804–50UG, Sigma Aldrich) diluted 1:1000 and a goat anti-mouse antibody conjugated with an Alexa-568 fluorophore (ab175473, Abcam) diluted 1:5000. Cell nuclei were counterstained with DAPI. Slides were imaged using a Nikon ECLIPSE Ti2 inverted microscope at 60× magnification. All images were processed using ImageJ software (National Institutes of Health).

### Quantification of FLAG-FOXI3 localization

Multiple images obtained at 60× magnification of FLAG-FOXI3 expressing cells were qualitatively categorized as showing isolated unclear staining, nuclear and cytoplasmic staining, or cytoplasmic staining with exclusion from the nucleus. A total of 250 to 400 cells were analyzed per FOXI3 genotype. A χ^2^ goodness of fit test was used to compare the cellular distribution of each missense variant with wild type, and a *P* value of <.05 was considered statistically significant.

## Results

**Figure 1:**
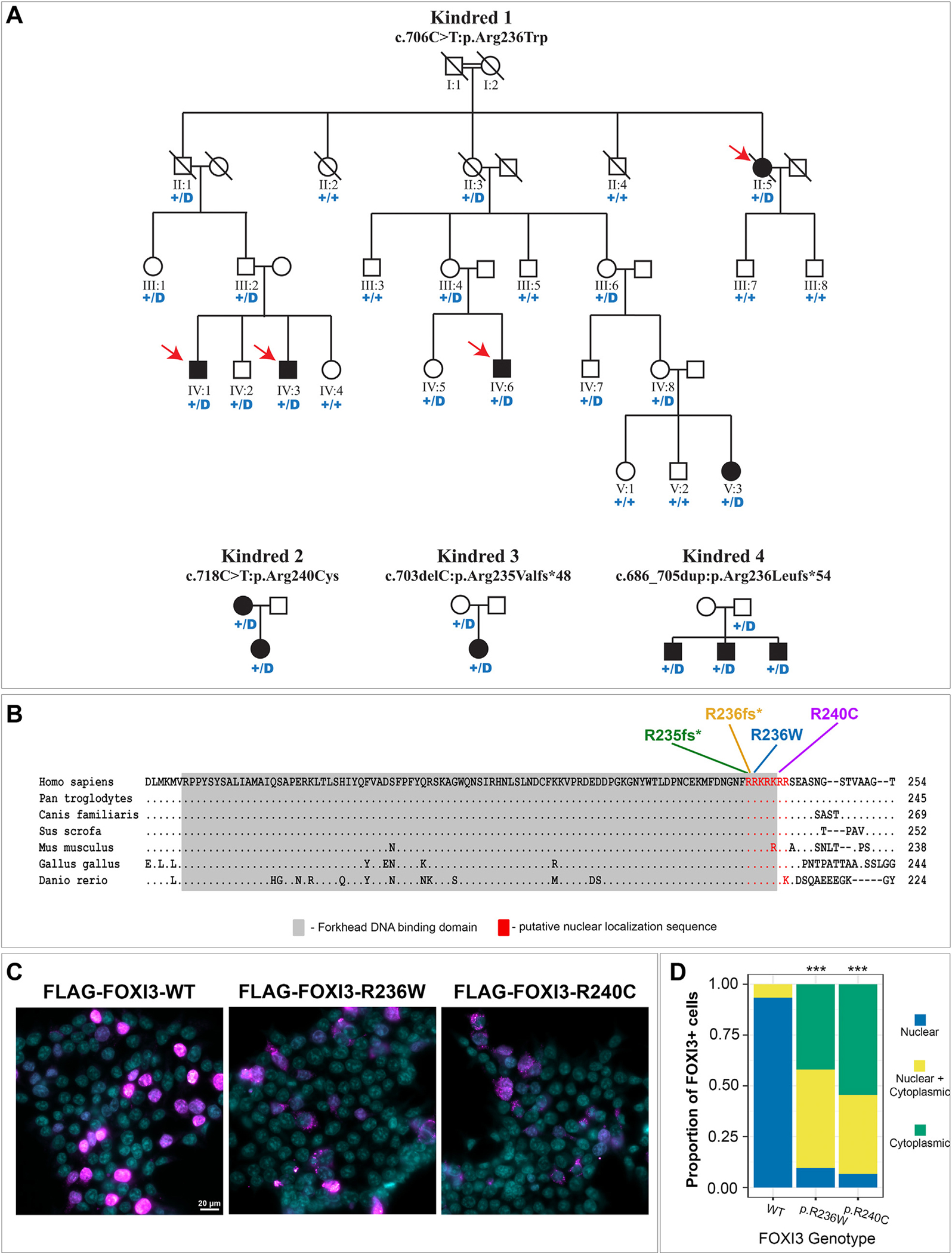
*FOXI3* variants in pedigrees with microtia and craniofacial microsomia. A. Pedigrees of 4 kindreds with *FOXI3* missense and truncating variants. “+” denotes the WT allele and “D” denotes the missense or truncating *FOXI3* variant indicated above the pedigree. Red arrows in kindred 1 indicate samples that underwent genome sequencing. B. Multiple sequence alignment of *FOXI3* protein sequence from vertebrate species. “.” indicates conservation with the human protein sequence. The locations of *FOXI3* variants in the nuclear localization sequence (NLS) are indicated above. The forkhead-DNA binding domain and NLS are highlighted. C. Immunocytochemistry of FLAG-FOXI3 (magenta) expressed in HEK293 cells. Cellular nuclei are counterstained with DAPI (cyan). All images are shown at 60× magnification. Scale bar = 20 μm. D. Quantification of FLAG-FOXI3 cellular localization in HEK293 cells from (C). A χ^2^ goodness of fit test was used to compare the distribution of each missense variant with WT. *** indicates a *P* value of <.001. WT, wild type.

**Figure 2:**
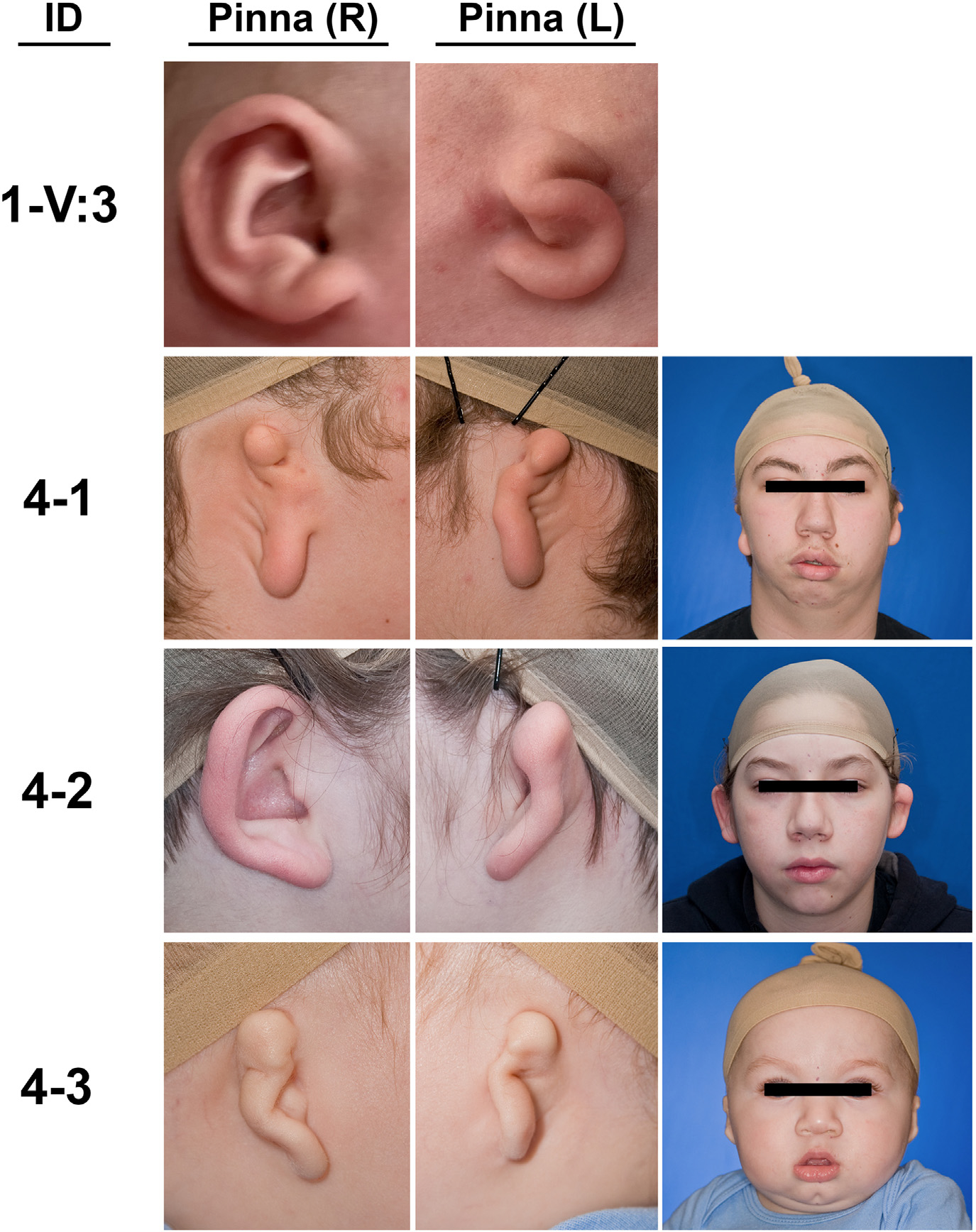
Clinical images of individuals with *FOXI3* variants. Clinical images show the phenotypic variability in kindreds with FOXI3 variants. Individual V:3 from kindred 1 (1-V:3) presents with unilateral (L) grade III microtia with aural atresia. Kindred 4 contains 3 affected siblings with variable phenotypes. Individual 4–1 presents with bilateral grade III microtia with atresia and bilateral (left side more affected than right) mandibular hypoplasia and facial tags (removed). Individual 4–2 presents with right grade I microtia with atresia and left grade II microtia with atresia. Individual 4–3 presents with bilateral grade III microtia with atresia and left mandibular hypoplasia. ID, patient identifier; L, left; R, right.

To identify novel loci for microtia-CFM, we studied a 5-generation kindred of Italian-American ancestry presenting with dominant microtia of unknown genetic cause (kindred 1, Figure 1A). The kindred included 5 affected individuals with unilateral or bilateral grade II/III microtia with or without aural atresia (Table 1; Figure 2), 4 of whom (II-5, IV-1, IV-3, IV-6) underwent GS through the GMKF Pediatric Research Program. To identify the causal variant, we first used ultrarare (gnomAD [v3] AF of <1.0 × 10^−5^ in non-Finnish Europeans) biallelic SNVs to identify haplotypes shared by the 4 affected individuals and mapped 7 shared genomic intervals, the largest of which was a 30 megabase pericentromeric region on chromosome 2 (Supplemental Figure 1). We evaluated damaging SNVs, indels, and genomic structural variants within and surrounding the shared genomic regions and identified only 2 shared missense variants in the genes *FOXI3* (chr2) and *PTCD3* (chr2). *FOXI3* is a transcriptional regular of ectodermal development with a critical role in murine pharyngeal arch and ear development^7^ and is associated with congenital ear malformations with variable penetrance in canine breeds with ectodermal dysplasia due to haploinsufficiency of *FOXI3.*^8,9^ In humans, a 2.5 megabase genomic deletion overlapping the *FOXI3* gene has been reported in a patient with microtia, aural atresia, and ipsilateral agenesis of the carotid artery,^9^ and recurrent deletions located on chromosome 2p11.2 that overlap the *FOXI3* gene have been reported in a DiGeorge syndrome like spectrum of phenotypes inclusive of hearing abnormalities and unilateral facial bone hypoplasia.^10^ On the basis of these data, we focused on *FOXI3* as the most likely candidate gene in the kindred. The *FOXI3* p.Arg236Trp (NM_001135649.1:c.706C>T) variant shared by affected individuals in kindred 1 alters an amino acid residue in a putative NLS that is highly conserved across vertebrate species (Figure 1B).^11^ This variant is absent from the gnomAD (v3) reference database and is predicted to be deleterious by several pathogenicity prediction tools (Combined Annotation Dependent Depletion score = 23.9, Polymorphism Phenotyping v2 score = 0.998). We genotyped a total of 24 individuals in kindred 1 through Sanger sequencing and confirmed that the additional affected individual (V-3) was heterozygous for the *FOXI3* p.Arg236Trp variant (Supplemental Figure 2). Of 15 genotype positive individuals, 5 had microtia, suggesting a penetrance of approximately 33% (Figure 1A). Parametric linkage analysis using a model accounting for variable penetrance showed statistically significant linkage between the *FOXI3* p.Arg236Trp variant and microtia with a LOD score of 3.33. We next evaluated the functional effect of the p.Arg236Trp missense variant by expressing wild-type and p.Arg236Trp FLAG-tagged FOXI3 in HEK293 cells and assessing nuclear localization through immunocytochemistry. Wild-type FOXI3 protein was almost exclusively localized to nucleus (93% of cells), whereas FOXI3 p.Arg236Trp exhibited abnormal nuclear and cytoplasmic localization in 49% of cells and exclusion from the nucleus with punctate cytoplasmic staining in 42% of cells (Figure 1C and D).

To determine the potential contribution of *FOXI3* damaging variants to the microtia-CFM spectrum of disorders, we analyzed exome sequencing and GS data from 413 additional kindreds, identifying 3 additional families with novel *FOXI3* damaging variants (Figure 1A; Table 1). This included an additional missense variant in the NLS (p.Arg240Cys) in a kindred with isolated microtia that was predicted to be deleterious (Combined Annotation Dependent Depletion score = 29.1, Polymorphism Phenotyping v2 score = 1.00) and similarly resulted in the disruption of FOXI3 nuclear localization in vitro (Figure 1C and D). Two frameshift variants were also identified. One (p.Arg235Valfs*48) was found in a proband with bilateral grade III microtia with atresia with severe bilateral mandibular hypoplasia and right macrostomia (Figure 2). The variant was inherited from an unaffected father (Figure 1A). The second was found in kindred with 3 affected children (p. Arg236Leufs*54) who each inherited the variant from an unaffected father (Figure 1A; Table 1). We compared the frequency of *FOXI3* LOF variants or damaging missense variants in the NLS identified in the microtia-CFM cohort to gnomAD and calculated a significant burden for each type of damaging variant in affected individuals (*P* = 1.7 × 10^−4^ for each, *P* = 5.8 × 10^−8^ combined).

## Discussion

In this article, we report linkage analysis of a large kindred and association studies in 2 cohorts with microtia-CFM spectrum phenotypes showing that damaging variants in *FOXI3* are responsible for a fraction of microtia-CFM cases. We studied 415 families with microtia/CFM, identifying 4 kindreds with damaging variants in *FOXI3*. The results implicate *FOXI3* variants as the second most common genetic cause of CFM behind *SF3B2* haploinsufficiency, causing approximately 1% of cases in our cohort.^3^ All cases identified had grade II/III microtia with 90% external auditory canal atresia and 27% of affected individuals showing CFM as well. Furthermore, 80% of affected individuals had bilateral microtia. Consistent with other monogenic causes of ear and mandibular malformation, kindreds with *FOXI3* damaging variants show variable expressivity and incomplete penetrance, including unilateral and bilateral cases in related individuals, as well as variable effects on the mandible. Whether the phenotypic variation is due to common or rare modifier alleles, environmental factors, or is stochastic in nature remains to be determined. Of note, each of the 4 variants identified were within the 7-amino acid NLS. Future studies will determine whether the effects of these variants are due to haploinsufficiency or novel protein function secondary to disruption of the NLS. Evaluation of *FOXI3* variants in additional cohorts with microtia-CFM will be necessary to fully characterize the phenotypic effects of *FOXI3* variants. We note that patients with CFM had LOF variants, whereas affected individuals with missense variants in the NLS showed only isolated microtia, which is often considered the mildest form of CFM. These findings may indicate dosage sensitivity of FOXI3, with hypomorphic missense variants more likely causing microtia and haploinsufficiency contributing to greater risk for more severe phenotypes (CFM). However, the coincident identification of both missense and truncating variants localized to the NLS and variable penetrance observed in families suggest the possibility that perturbed cellular localization of FOXI3 and other factors contribute to craniofacial phenotypes.

FOXI3 primes preplacodal ectoderm for inductive signals that coordinate development of the otic and epibranchial placodes that give rise to the inner ear, and *FOXI3* haploinsufficiency has been shown to increase ectodermal apoptosis in mice.^12^ In addition, *FOXI3* mutant mice also show absence of the mandibular division of the trigeminal ganglion.^12^ The trigeminal nerve courses through the mandible, which is notable given that several patients in this series present with mandibular hypoplasia, a characteristic feature of CFM. Whether malformation of the mandibular branch of the trigeminal nerve contributes to mandibular hypoplasia in patients with CFM remains uncertain. However, ectodermal disruption is a likely cause of microtia-CFM in patients with *FOXI3* damaging variants. Mice with genetic loss of *Foxi3* show complete absence of the external and internal ears.^7^ Cranial neural crest cells migrate to the developing pharyngeal arches but then undergo apoptosis, suggesting a role for *Foxi3* in neural crest survival.^7^ Neural crest cells are likely disrupted in patients with other prevalent monogenic causes of CFM^3^ and are also likely affected by a common disease risk haplotype conferring ancestry-associated microtia-CFM risk.^4^ These results support disruption of neural crest by the absence of functioning ectodermal *FOXI3* as a likely mechanism of microtia-CFM pathogenesis.

These data support genetic testing for *FOXI3* variants in patients with grade II/III microtia with atresia, with the highest anticipated diagnostic yield in bilateral and familial cases. These findings contribute to a growing body of evidence supporting neural crest disruption in CFM and add *FOXI3* to the list of identified monogenic causes of microtia-CFM spectrum disorders that includes *SF3B2*, *OTX2* duplication, and *HOXA2* among other less prevalent causes. Sequencing substantially larger cohorts to identify additional CFM loci will be necessary to elucidate additional genes and pathways that contribute to the microtia-CFM spectrum of disorders.

## Supplementary Material

Supplemental Info

## Figures and Tables

**Table 1:** Clinical characteristics of individuals with pathogenic *FOXI3* variants

Proband ID	1-II:5	1-IV:1	1-IV:3	1-IV:6	1-V:3	2	3	4–1	4–2	4–3

Inheritance	Unknown	Paternal	Paternal	Maternal	Maternal	Maternal	Maternal	Paternal	Paternal	Paternal
Nucleotide change (NM_001135649.1)	c.706C>T	c.706C>T	c.706C>T	c.706C>T	c.706C>T	c.718C>T	c.703delC	c.686_705dup	c.686_705dup	c.686_705dup
Amino acid change	p.Arg236Trp	p.Arg236Trp	p.Arg236Trp	p.Arg236Trp	p.Arg236Trp	p.Arg240Cys	p.Arg235Valfs*48	p.Arg236Leufs*54	p.Arg236Leufs*54	p.Arg236Leufs*54
Sex	Female	Male	Male	Male	Female	Female	Female	Male	Male	Male
Facial asymmetry	–	–	–	–	–	–	Bilateral mandibular hypoplasia	Bilateral mandibular hypoplasia	–	Left mandibular hypoplasia
Coloboma	–	–	–	–	–	–	–	–	–	–
Lateral oral cleft	–	–	–	–	–	–	R macrostomia	–	–	–
Ear abnormalities	Grade 3 microtia (left) with atresia	Grade 2 microtia (bilateral)	Grade 3 microtia (bilateral) with atresia	Grade 3 microtia (bilateral) with atresia	Grade 3 microtia (left) with atresia	Grade 3 microtia (bilateral) with atresia	Grade 3 microtia (bilateral) with atresia	Grade 3 microtia (bilateral) with atresia	Grade 1 microtia with atresia (right), grade 2 microtia with atresia (left)	Grade 3 microtia (bilateral) with atresia
Skin tags	–	–	–	–	–	–	–	Left facial tag	Sinus tract from auricular pit along posterior helical border	–
Other birth defects	–	–	–	–	–	–	–	Submucous cleft palate and bifid uvula	Bifid uvula	–
Ancestry	European	European	European	European	European	Latin American	European/Hispanic	Amerindigineous/Native Alaskan	Amerindigineous/Native Alaskan	Amerindigineous/Native Alaskan

*ID*, identifier; *R*, right.

## Data Availability

De-identified genome sequencing data sets sequenced as part of the NIH Gabriella Miller Kids First Program are available via controlled access from database of Genotypes and Phenotypes (accession numbers phs002172 and phs002130) and at kidsfirstdrc.org. All other data are available upon request.
