# Supplementary material for: Damaging variants in FOXI3 cause microtia and craniofacial microsomia
Source: Genet Med. Author manuscript; Available in PMC 2023 Jan 30. (PMC9885525; doi:10.1016/j.gim.2022.09.005)
Supplement: Supplemental Info [file NIHMS1864558-supplement-Supplemental_Info.pdf]

Supporting Information for '**Damaging variants in *FOXI3* cause microtia and craniofacial microsomia**'

This appendix contains Supplemental Figures S1-2.

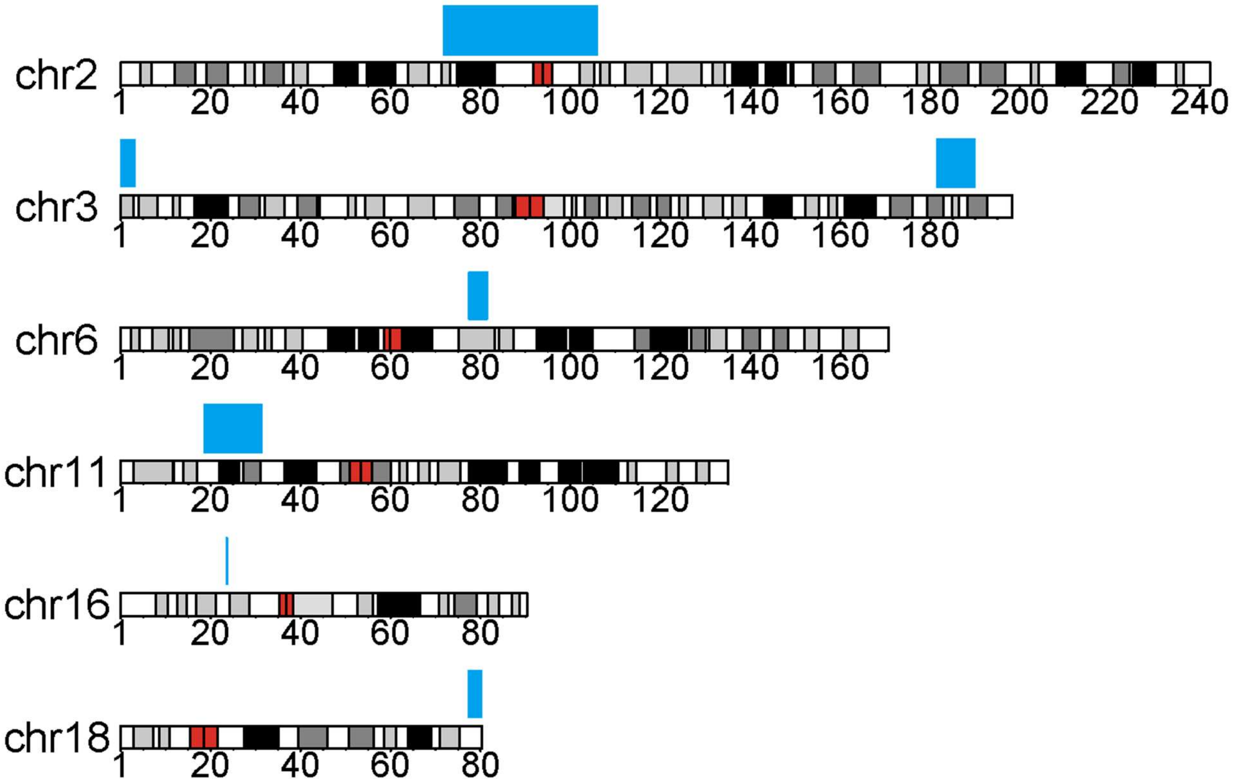

Figure S1. Mapping shared haplotypes in Kindred 1 using rare variants. Locations of shared heterozygous haplotypes in 4 individuals from Kindred 1 (II-5, IV-1, IV-3, IV-6) with WGS are shown in blue. Coordinates are in hg38.

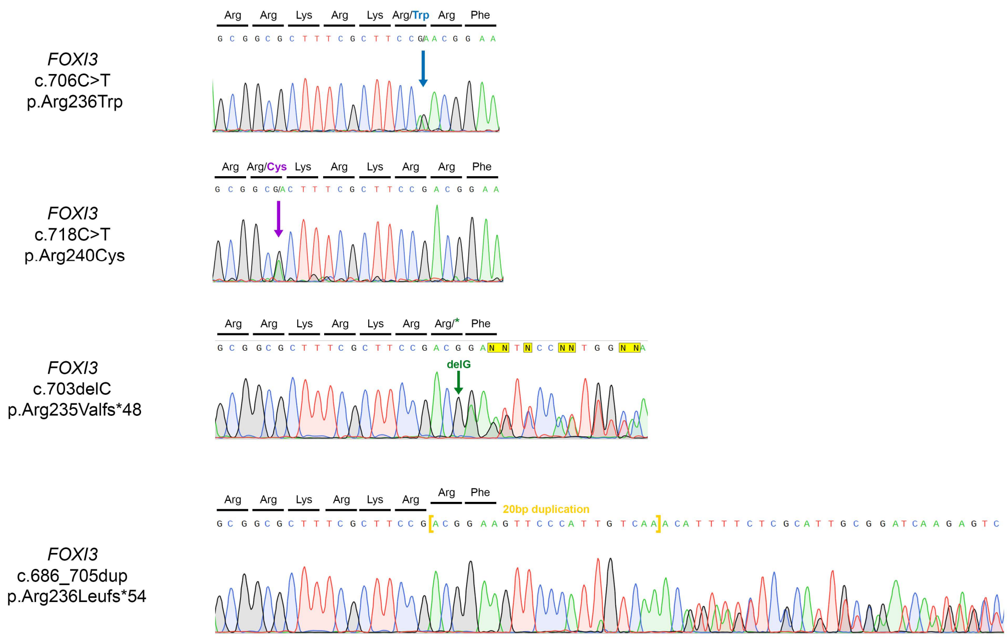

Figure S2. Confirmation of *FOXI3* variants by Sanger Sequencing.
